# Hepatoprotective Potential of Herbal Medicine

**DOI:** 10.1155/2015/536564

**Published:** 2015-08-04

**Authors:** Ravirajsinh Jadeja, Ranjitsinh V. Devkar, Srinivas Nammi

**Affiliations:** ^1^Division of Gastroenterology and Hepatology, Department of Medicine, Medical College of Georgia, Georgia Regents University, Augusta, GA 30912, USA; ^2^Division of Phytotherapeutics and Metabolic Endocrinology, Department of Zoology, Faculty of Science, The MS University of Baroda, Vadodara, Gujarat 390002, India; ^3^School of Science and Health, University of Western Sydney, Sydney, NSW 2751, Australia; ^4^NICM-Centre for Complementary Medicine Research, University of Western Sydney, Sydney, NSW 2751, Australia

Occurrence of various types of hepatic ailments has been on the rise and has resulted in significant increase in morbidity and mortality worldwide. Viral hepatitis, alcoholic/nonalcoholic fatty liver disease, liver fibrosis, cirrhosis, hepatocellular carcinoma, and drug-induced liver injury are of major health concerns claiming millions of lives worldwide. Pharmaceutical drugs are often associated with liver injury and hence provide limited benefits in treating liver ailments. Use of herbal medicine to treat liver ailments has been mentioned in various ancient literature texts and a considerable number of medicinal plants have been evaluated using preclinical and clinical studies as hepatoprotective agents. Nevertheless, more rigorous studies are still required to screen and evaluate the use of herbal medicines in treating various liver diseases.

This special issue is a collection of five articles describing the use of herbal medicines against various liver diseases. An article by A. Sadeghipour et al. described lipid lowering effect of pomegranate (*Punica granatum* L.) peel extract in high fat diet fed rats. Authors also recommended that plant should be considered an excellent candidate for future studies on dyslipidemia. There are two research articles describing beneficial role of herbal medicines against alcoholic induced liver injury. P. Lodhi et al. showed that aqueous extract of* Camellia sinensis* (green tea) was able to ameliorate ethanol induced acute liver damage. An interesting article by L. An and F. Feng described network pharmacology-based antioxidant effect of Zhi-Zi-Da-Huang decoction, a classic traditional Chinese medicine (TCM) formula for alcoholic liver disease. It was concluded that network pharmacology is a useful tool for exploring the potential mechanism of action of TCM formula and new active ingredients. The only clinical study as a part of this special issue by M. V. Patel et al. showed benefits of a complex multiherbal regimen based on Ayurvedic medicine for the management of hepatic cirrhosis complicated with ascites. Silymarin is perhaps the most widely used compound for liver diseases. A study by J.-P. Wu et al. demonstrated that silymarin administration accelerates liver regeneration after partial hepatectomy.

Collectively this special issue provides recent updates on the use of herbal medicine in treating acute and chronic liver diseases. We hope that this special issue will be useful to scientific fraternity.

